# Socially-Central Zebrafish Influence Group Behavior More than Those on the Social Periphery

**DOI:** 10.1371/journal.pone.0055503

**Published:** 2013-01-31

**Authors:** Cuauhcihuatl Vital, Emília P. Martins

**Affiliations:** 1 Department of Veterinary Sciences and Animal Husbandry, Universidad Autónoma de Ciudad Juarez, Ciudad Juárez, Chihuahua, México; 2 Department of Biology & Center for the Integrative Study of Animal Behavior, Indiana University, Bloomington, Indiana, United States of America; Cajal Institute, Consejo Superior de Investigaciones Científicas, Spain

## Abstract

Certain individuals are more effective than others at using individual experience to impact group behavior. Here, we tested whether pre-training of zebrafish that are at the focal central of social group dynamics (“Key” fish) has a stronger positive impact on group performance than does pre-training of less central (“Non-Key”) fish. We used very short observation periods and social network statistics to identify Key and Non-Key individuals, trained these fish to respond to an aversive stimulus, and then measured group performance after returning these now-experienced fish to a social setting. Although Key and Non-Key fish evaded the stimulus equally quickly as individuals, groups with experienced Key fish escaped the aversive stimulus more quickly than did groups with experienced Non-Key fish. The impact depended on genetic background: PN zebrafish on the social extremes (more often males) influenced the group's baseline response to the aversive stimulus, whereas experienced Scientific Hatcheries' zebrafish (both males and females) influenced the change in response over repeated trials. These results suggest that social roles are an important feature of information transfer across a group, and set the stage for future research into the genetic and evolutionary basis of social learning.

## Introduction

Learning indiscriminately from other members of the group may not be adaptive (see reviews by [1], [2], [3]). Instead, animals may do better by using phenotypic characteristics to identify more successful, older, or more skilled individuals (e.g., [4], [5], [6]). Alternatively, social dynamics may guide the choice of leaders and learning models (e.g., [7]). Some individuals may have greater influence on group behavior simply because they are more often in physical proximity to others or are able to attract social attention better than others (e.g., [8], [9], [10]). Selection may even encourage individual animals to actively choose and form groups that offer the best context for their particular strategies (i.e., social niche construction, [11], [12]). Here, we ask whether zebrafish at the center of their social networks have a greater impact on group behavior than do less socially-connected individuals.

We use social network statistics, popular tools for measuring social behavior that offer unprecedented insight into group dynamics (e.g., [13], [14], [15]). Specifically, we use social network statistics to identify “Key” individuals, those that are most central to the group because they interact readily with other animals (e.g., [16], [17]). Key individuals have the opportunity to influence group behavior in several different ways [7]. By engaging in frequent social interactions, Key animals enhance information flow across sub-groups [18], [19] and increase social stability [20]. They may serve as learning models because of physical proximity (e.g., [21], [22]) or social status [23], [24]. Key animals may also impact group behavior by actively determining the direction of group movement (e.g., [25], [26]).

Many species of fish gain information about their environment through interactions with shoal mates (see review by [27]). Our study uses zebrafish, small cyprinids found in shoals of 4–10 fish in shallow lakes and streams throughout India and Bangladesh [28], [29]. In particular, many species of fish, including zebrafish [30], learn about aversive experiences such as predators from conspecifics (see reviews by [31], [32]). As in other vertebrates, fish social interactions lead to learning and can be influenced by attributes such as familiarity [33] and sex [34] of shoal mates, as well as, shoal size [35]. The specific type of information being conveyed can also be an important predictor of the long-term stability of the socially-learned information (e.g., [36], [37]). Here we ask whether individual social attributes also impact group learning, using zebrafish as a model.

We recently found that experimental removal of a Key individual impeded a zebrafish group's ability to move towards food [17]. These early results also suggested that zebrafish from the domesticated Scientific Hatchery (SH) strain have evolved a more flexible use of social roles than zebrafish from the more recently-established PN strain, with individual SH fish switching roles more fluidly in response to experimental perturbation. Here, we expand on these earlier results by asking whether Key zebrafish are more effective at influencing the behavior of their social groups and whether strain differences or sex impact that influence on group behavior. By experimenting with zebrafish, a biomedically-important model organism, we also contribute by further developing the use of social network statistics based on very short observation periods, and thereby provide tools for future studies of the genetic and developmental bases of social behavior (e.g., [38], [39]).

## Methods

### Ethics statement

All experiments comply with current laws of the United States of America and with the Animal Care Guidelines of Indiana University (BIACUC protocol approval #: 07-074).

We used a total of 31 groups of zebrafish, each containing 2 males and 2 females of roughly the same body length. Shoals of 4 fish are probably small for zebrafish in the wild, but have been used effectively in experimental contexts (e.g., [40], [41]). All of our subject fish were housed in standard conditions from hatching to about 4 months of age (single-strain groups, 18.9l aquaria, 24–27°C, abundant food, 13L∶11D light cycle, filtered and aerated water). Subjects from the current experiment were different individual fish bred from the same two strains in Vital & Martins [17]. We formed 14 groups of fish from the Scientific Hatcheries (SH) line (a domesticated, but genetically-outbred line that has been reared since the 1990's in high-density conditions), and 17 groups of fish from the more recently-established PN strain (established in 2007 with wild-caught fish from an oxbow lake in West Bengal, India, but maintained in our lab for 2–3 generations prior to the current experiments).

**Figure 1:**
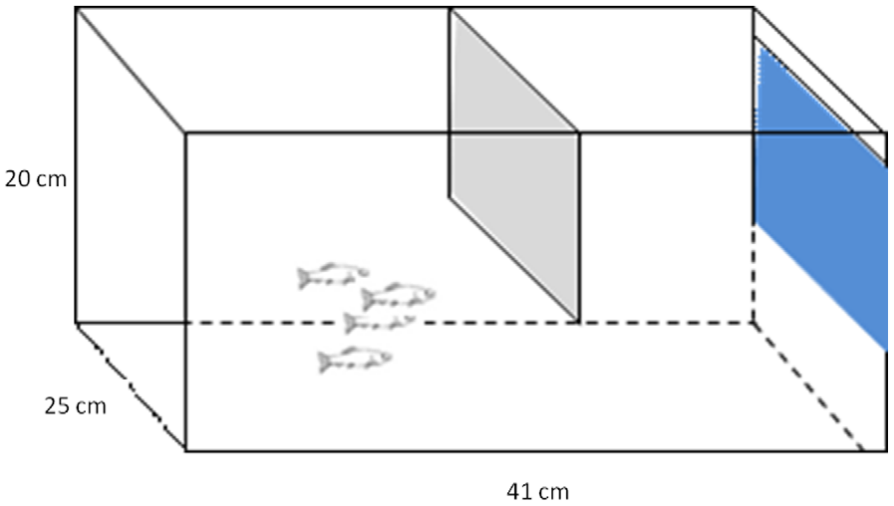
The testing tank arena used for this study. We used 5 gallon aquaria (18.9 L) which are 16″×8″×10″(41×20×25 cm).The testing tank was partially divided by a central plastic plate that left enough room at the bottom to allow fish to swim back and forth between the two sides.

We visually isolated the experimental groups from each other by placing opaque barriers between the aquaria. Each aquarium was further partially divided by a central plastic plate that left enough room at the bottom to allow fish to swim back and forth between the two sides (Fig. 1). We began behavioral assays (below) two weeks after forming these groups. To minimize stress, we assayed social behavior in the home aquaria, with a single human observer (C.V.) conducting observations from behind a black curtain blind. We have found this approach to be highly repeatable (>94% even when data are scored by different observers) [17].

### Experimental protocol: Identifying Key and Non-Key individuals

We were easily able to distinguish the four individuals in each group using subtle differences in natural striping patterns and body shape (there were no major differences such as male barbs). As in Vital and Martins [17], we conducted 4-min assays of social behavior to identify individuals in each group playing potentially distinct social roles. Specifically, one investigator observed each fish in the group continuously for a 1-min focal animal sample, recording the identity of every other fish that came within 2 body lengths of the focal individual and whether it was approached by or approached the focal individual (directed near-neighbor points). Others (e.g., [42]) use a distance of 4 body lengths to define social interactions in the wild. We use a more conservative measure of two body lengths for this laboratory study because of the space limitations. The observer then repeated this procedure with the three other fish in the same group, collecting at least 10 near-neighbor points for each subject (as recommended for social network statistics, [43]).

Also as in Vital & Martins [17], we used these directed near-neighbor data to estimate Information Centrality (IC, [44]) for each fish, identifying the individual with the highest value of IC as a “Key” fish and the individual with the lowest estimated IC value as a “Non-Key” fish. Thus, the Key fish is the group member that most often remained in close proximity to most of the other fish, whereas the Non-Key fish was the individual least likely to be within two body lengths of other fish in the group or that remained in the proximity of only one other fish. Despite the very short observation periods, we found previously [17] that estimates of social dynamics based on these calculations are both reliable and repeatable even when taken several days apart.

### Experimental protocol: Training Procedure

Once we identified Key and Non-Key fish for each group, we used a hand net to remove one individual fish from each group (to be trained in isolation before being returned to their groups 24 h later). We isolated the Key fish from roughly half of the groups (9 PN and 8 SH groups) and the Non-Key fish from the remaining half (8 PN and 6 SH groups). We placed these isolated fish individually into semi-divided test arenas identical to their home tanks (Fig. 1), and allowed them to rest overnight. On the subsequent day, we presented the isolated fish with three treatment sessions (see below): once between 9am–10am, once between 12pm and 1pm, and once between 3pm and 4pm. We then returned the now-experienced fish to their original groups, leaving them to recover from the stress of the adverse stimulus for two days before further testing. After those two days, we assayed behavior of each group of 4 fish using the same series of three treatment sessions on the group as a whole.

At the end of each training session, the investigator placed a blue index card against the glass to mark one or the other side of the semi-divided experimental arena (chosen at random with equal probabilities). About 2 h later, the experimenter began the next session by attracting the subject's attention by lightly tapping the glass near the lower center of the arena, at the point where the subject fish could swim beneath the partial barrier between the two sides of the arena. Subject fish usually oriented and moved towards this tapping stimulus, without showing overt signs of fear or startle. The experimenter then lowered a 10-cm wooden stick into the blue-marked side of the aquarium, moving the stick back and forth slowly in a stereotyped fashion for 8 seconds or less if fish left the side with the stimulus. Subject fish presented with this stimulus quickly swam away from the stick, under the barrier to the opposite side of the aquarium. We recorded the total time (s) from the initial lowering of the stick to when the subject fish (single individual or all four fish in a group) moved to the opposite side of the arena. We then determined whether or not to move the blue card to the other side of the arena (chosen at random with equal probabilities), waited 30 min, and repeated the entire process. The two measures were quite similar to each other, so we averaged the times for the two parts of each session and use these averages as the units for our statistical analyses.

### Statistical Analyses

We used repeated-measures ANOVA to test the effects of social role (Key or Non-Key) on escape time of the isolated subject fish across the three training sessions, including also terms to test the effects of strain (PN or SH), sex (male or female), and their interactions. We used a similar repeated-measures analysis to test the effect of the same qualities of the isolated fish (social role, strain, sex, and their interactions) in explaining variation on group escape time after the experienced fish had been returned to its group. We conducted all analyses in SAS [45], and used residual analyses to confirm the usual normality and homoscedasticity assumptions.

## Results

### Strain and sex, but not social role, predict individual escape from an adverse stimulus

**Figure 2:**
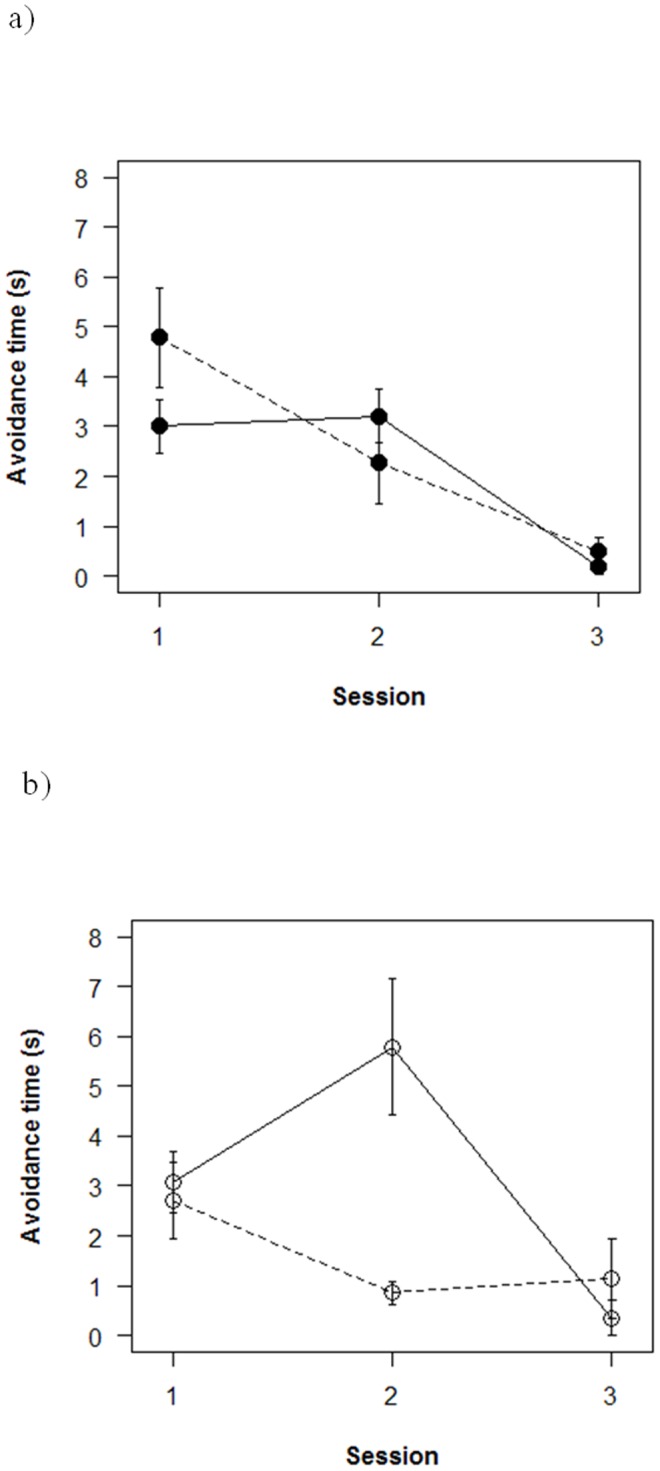
Individual zebrafish decreased escape time from an aversive stimulus across three sessions. 2a) Female SH zebrafish (dashed lines) decreased escape time by the second session, whereas female PN zebrafish (solid lines) generally took until the third session to evade effectively. 2b) Male zebrafish showed a steady improvement in response time regardless of strain, leading to a sex×strain interaction effect during the second training session. Error bars are one standard error.

In our comprehensive repeated-measures analysis, we found no significant evidence for most interactions between social role, sex, and strain of tested fish (P>0.05). Individual zebrafish (Fig. 2) escaped from the stick more quickly after the three experimental sessions, decreasing their escape time from a mean of 3.4 s±0.37 (one S.E.) on the first session to a mean of 0.5 s±0.21 on the third session (time profile effect: Wilk's λ = 0.35, F = 20.1, df = 2, 22; p<0.0001). Key and Non-Key fish did not differ significantly in terms of their individual escape times across the three sessions (p>0.8). However, SH fish (dashed lines in Fig. 2) escaped more quickly by the second session (S2), whereas PN fish (solid lines in Fig. 2) showed faster escape times only at the third session (S3; strain effect on time profile: Wilk's λ = 0.58, F = 7.9, d.f. = 2,22, p<0.002). This strain difference was most dramatic during the second session when individual PN fish took an average of 4.3 s±0.62 to evade the moving stick in comparison to 1.6 s±0.53 for SH fish (strain effect: F = 12.2, d.f. = 1, 23, p<0.002). We found a significant difference in size of Key and Non-Key individuals (p = 0.05, t-test) with Key individuals being larger (2.8 cm) than Non-Key fish (2.6 cm).

Although there was no clear sex difference in which individuals were identified as Key or Non-Key fish, sex differences in behavior were intertwined with strain differences. A few more male than female PN (10 M vs 7 F) fish exhibited extreme behavior and were thus removed from their groups as Key or Non-Key fish, whereas there was no comparable sex difference in SH fish (7M and 7F were isolated as Key or Non-Key). In addition, individual female PN fish (solid lines, Fig. 2a) were slower than were PN males (solid lines, Fig. 2b) to avoid the aversive stimulus during the second session (S2), whereas there was less sex difference in SH fish (dashed lines, Fig. 2ab). This resulted in a significant sex×strain interaction effect during this middle session (S2: F = 5.2, d.f. = 1,23, p<0.04), and also between subjects overall (F = 5.7; df = 1,23; p<0.03). No other interactions or main effects were statistically significant (P>0.05, results not shown).

### Groups with experienced Key individuals escaped faster than did groups with experienced Non-Key individuals

**Figure 3:**
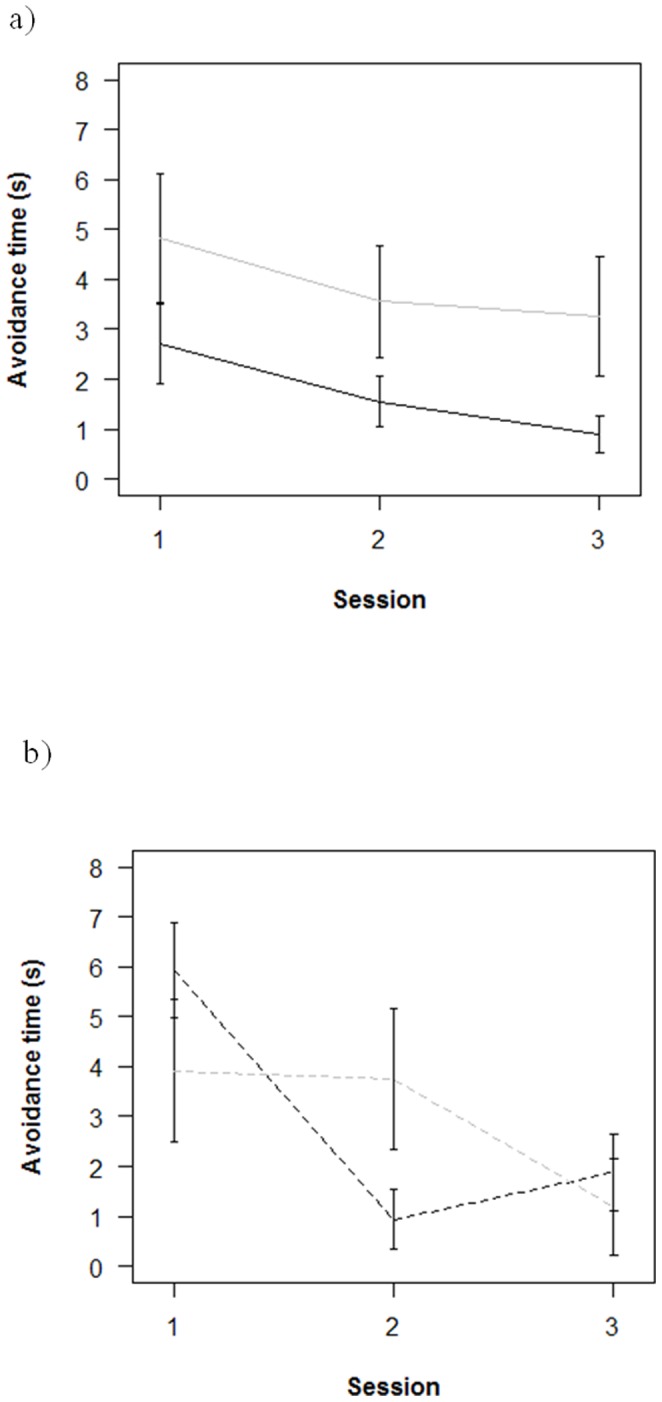
Groups with experienced Key individuals (black lines) were better able to avoid the stimulus. Groups with experienced Key individuals were better at avoiding the stimulus than were groups in which the experienced fish was a non-Key fish (gray lines). 3a) In PN fish, the effect of social role was in terms of faster escape for groups with experienced Key fish (solid black line) than for groups with experienced non-Key fish (solid gray line) at all three sessions. 3b) In SH fish, the difference was in terms of faster improvement: by the second session (S2) for groups with experienced non-Key fish (gray dashed line), but only by the third session (S3) for groups with experienced non-Key fish (gray solid line). Error bars are one standard error. Data are for the same trials as in Fig. 4, but combining data from males and females.

Social role of the single experienced fish had an impact on group escape behavior (between-subjects effect: F = 7.9; df = 1,23; p = 0.01), but the effect was complicated by an interaction with strain (F = 11.3; df = 1,23; p<0.003). Groups of zebrafish responded more quickly to the stick after repeated sessions (Fig. 3), leading to a significant effect of session on escape response (time profile effect: Wilk's λ = 0.68, F = 5.2, d.f. = 2, 22; p<0.02). PN groups (Fig. 3a) with experienced Key individuals (black line) avoided the aversive stimulus roughly twice as quickly at every session as did PN groups with experienced Non-Key individuals (gray line). In contrast, there was no clear difference in the baseline escape behavior of SH groups with experienced Key or Non-Key individuals (Fig. 3b). As in the individual fish sessions, SH groups that had experienced Key individuals (black line) improved performance more quickly (by the second session, S2) than did SH groups with experienced non-Key individuals (gray line: by the third session, S3, Fig. 3b). Overall, the effect of social role was significant as a main effect for the second session (S2: F = 8.2; df = 1,23; p<0.01), and as an interaction with strain during the first (F = 6.8; df = 1,23; p<0.02) and third (F = 5.1; df = 1,23; p = 0.03) sessions.

### Sex differences were also important

**Figure 4:**
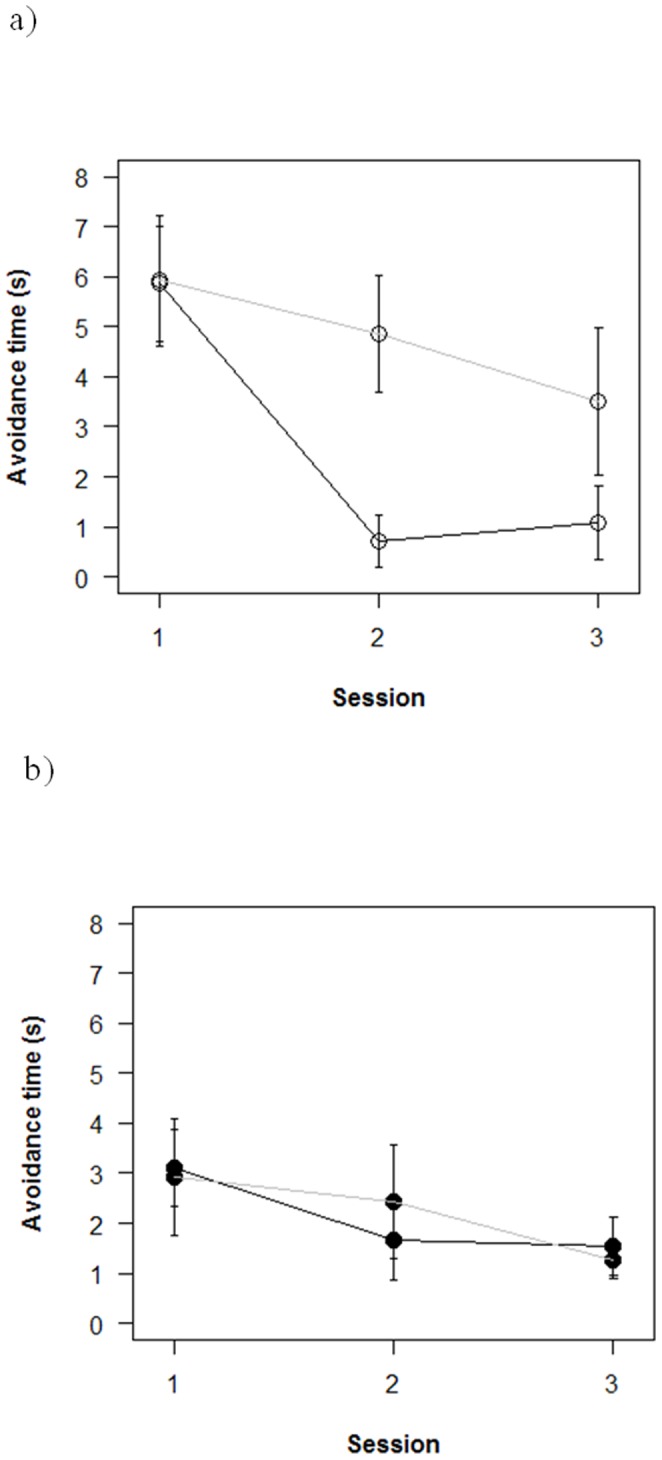
Sex effect of experienced fish (key and non-key) differs according to genetic background. 4a) Groups with experienced females avoided the aversive stimulus slowly at the first session, but improved performance more quickly when the experienced female was a Key fish (black line) as opposed to a non-Key fish (gray line). 4b) Groups with experienced males avoided quickly from the first trial, and showed little change in performance across the three sessions leading to a significant interaction effect. Error bars are one standard error. Data are for the same trials as in Fig. 3, but combining data from PN and SH strains.

The effect of social role was also complicated by sex differences in escape behavior, with experienced females playing an especially important role (Fig. 4). Groups with experienced females (Fig. 4a) had slower initial escape times (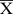 = 3.0 s±0.64) than did groups with experienced males (Fig. 4b, 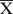 = 5.9 s±0.84), leading to a significant between-subjects effect of sex (F = 15.2; df = 1,23; p<0.001). Groups with experienced Key females improved performance quickly, having the fastest escape time of all groups by the second and third sessions (Fig. 4a, black line). Groups with experienced Non-Key females (Fig. 4a, gray line) also improved escape, but even at the third session remained slower on average than other types of groups. In contrast, the social role of experienced males had little effect on group escape behavior (Fig. 4b), leading to a significant between-subjects social role X sex interaction effect (F = 7.0; d.f. = 1,23; p<0.02).

We found no significant evidence for other possible interactions (e.g., between sex, social role, and strain) or main effects (P>0.05, results not shown).

## Discussion

Our results show that individuals that play a central social role can influence group behavior more effectively than individuals on the social periphery. Key zebrafish (individuals that interacted frequently with most of the other fish in their groups) translated individual prior experience into improved group performance more effectively than did Non Key fish. Intriguingly, the mechanism underlying this pattern has evolved: the effect was immediate for zebrafish from a recently-established strain (PN), whereas domesticated zebrafish (SH strain) influenced their groups by speeding learning over repeated sessions. There was also a sex difference: male fish influenced group movement regardless of social position, whereas socially-central females had a much stronger impact than did peripheral females. Through this experiment, we also confirm the utility of social network statistics based on very short periods of behavioral observation, relatively small numbers of fish groups, and applied to aversive as well as appetitive contexts. Despite these limitations of our data, our results set the stage for future research into the behavioral, genetic and physiological mechanisms underlying the evolution of social roles.

Our findings support the idea that group motion is, in part, an indirect consequence of social relationships. Others have found that sheep move readily in the direction of any animal leaving the spatial core of the group [46], and training of a single shiner to prefer a particular location causes the entire fish shoal to spend more time there [47]. In human networks, single individuals have greater impact on information flow when the group is characterized by direct and repeated social interactions [48]. Our finding that Key fish have a stronger impact on group behavior extends this theme by showing that in structured zebrafish groups, individuals in socially-central positions have a greater indirect impact than those on the social periphery. Additional studies are needed to determine whether other socially-central animals, such as “policing” macaques that act overtly to improve social stability [20], also influence group learning or the direction of group movement, and whether they do so by leading in front of the moving group or by herding the other fish from behind.

Others have also shown that individuals with certain phenotypic characteristics, physiological states, or previous knowledge can influence group movement. For example, individual barnacle geese that consistently approach novel objects are also the ones likely to be on the leading edge of their flocks [49]. In non-human primates, leaders are characterized by a combination of attributes such as dominance and physiological state (e.g., [50]). In humans, individuals that have more “information production” and “political power” than other group members are more likely to impact group behavior [48]. Although we did not detect any major morphological differences between zebrafish in different social roles in the current study, such differences may be subtle. We know that both genetic strain and physiological state can impact the speed at which zebrafish groups evade a predator or aversive stimulus [51] and the overall social dynamics of zebrafish groups [17]. Frequency-dependent selection can maintain groups with divergent personality features to ensure an optimal number of leaders in each group [11], [52].

Male and female zebrafish are so similar in terms of morphology and behavior that sex differences in zebrafish research studies are often negligible or ignored (e.g., [53]). As explained above, here we find that male zebrafish influenced group movement more immediately, whereas the impact of females is on enhanced learning over several days. In guppies, novel foraging information spreads at a significantly faster rate through subgroups of females than through subgroups of males [54]. Other studies have also found that the sex of individuals engaging in different types of social behavior strongly influences group performance and social dynamics. For instance, juvenile males living in close contact to adult females exhibit a different song development pattern than do juvenile males living in close contact with juvenile females [55]. In zebra finches, sex of the demonstrator influences social learning [56] and sex plays a major role in other aspects of social personality (Schuett and Dall 2009).

Although social engagement and individual strengths can both be important mechanisms by which Key fish influence group movement, our results also suggest that the relative importance of these two mechanisms can evolve. Using different fish and an aversive rather than an appetitive context, our current results confirm and expand earlier suggestion of differences between PN and SH zebrafish strains [17]. In that earlier study, individual fish from the recently-wild PN strain appeared to take on fixed social roles, as if social role were determined by phenotypic or behavioral characteristics (e.g., sex or personality) that are not easily changed. More male than female PN fish were identified in extreme social roles (Key or Non-Key), implicating sex as an important determiner of social position. Here, we find also that the impact of PN Key fish (mostly males) on group movement is immediate, as if they are directly influencing the group, perhaps leading them away from the aversive stimulus. In contrast, zebrafish from the domesticated SH strain switched quickly between roles when disturbed, and there was no difference in the proportion of males and females taking on those roles [17]. In the current study, the impact of SH Key fish was in terms of improving performance across three training sessions, as if influence of SH Key fish is due to their social influence, through enhancing group learning and coordinated movement.

Because of the abundance of genetic tools available for studying zebrafish, strain differences of this sort can provide an unparalleled opportunity for understanding the genetic basis underlying evolution of vertebrate social roles and learning. Further comparisons of zebrafish collected recently from different wild populations would also be useful in identifying selective factors guiding social evolution. Other studies that vary group size, that carefully track individual movement, and that look for more detailed relationships between centrality measures and group movement would also contribute to our understanding of social facilitation.. Fortunately, it appears that even very short observation periods are sufficient to identify individuals playing different roles in a zebrafish group and likely to have different impacts on future group learning and movement. Whether the impact of these animals is by social facilitation or individual characteristics remains to be seen.

## References

[1] Boyd R, Richerson PJ (1995) Why does culture increase human adaptability? Ethology and Sociobiology 16: 125–143.

[2] Giraldeau LA, Valone TJ, Templeton JJ (2002) Potential disadvantes of using socially acquired information. Philosophical Transactions of the Royal Society B-Biological Sciences 357: 1559–1566. 10.1098/rstb.2002.1065 PMC1693065 12495513

[3] Laland KN (2004) Social learning strategies. Learning & Behavior 32: 4–14. 15161136 10.3758/bf03196002

[4] Horner V, Proctor D, Bonnie KE, Whiten A, de Waal FBM (2010) Prestige affects cultural learning in chimpanzees. Plos One 5: 5. 10.1371/journal.pone.0010625 PMC2873264 20502702

[5] Forsman JT, Seppanen JT (2010) Learning what (not) to do: testing rejection and copying of simulated heterospecific behavioural traits. Animal Behaviour 81: 879–883.

[6] Morand-Ferron J, Cole EF, Rawles JEC, Quinn JL (2011) Who are the innovators? A field experiment with 2 passerine species. Behavioral Ecology 22: 1241–1248.

[7] Coussi-Korbel S, Fragaszy DM (1995) On the relation between social dynamics and social learning. Animal Behaviour 50: 1441–1453.

[8] Scheid C, Range F, Bugnyar T (2007) When, what, and whom to watch? Quantifying attention in ravens (*Corvus corax*) and jackdaws (*Corvus monedula*). Journal of Comparative Psychology 121: 380–386. 18085921 10.1037/0735-7036.121.4.380

[9] Schwab C, Bugnyar T, Schloegl C, Kotrschal K (2008) Enhanced social learning between siblings in common ravens, *Corvus corax* . Animal Behaviour 75: 501–508. 25948875 10.1016/j.anbehav.2007.06.006 PMC4417712

[10] van de Waal E, Renevey N, Favre CM, Bshary R (2010) Selective attention to philopatric models causes directed social learning in wild vervet monkeys. Proceedings of the Royal Society of London, Series B: Biological Sciences 277: 2105–2111. 20236972 10.1098/rspb.2009.2260 PMC2880145

[11] Bergmüller R, Taborsky M (2010) Animal personality due to social niche specialisation. Trends in Ecology & Evolution 25: 504–511. 20638151 10.1016/j.tree.2010.06.012

[12] Saltz JB, Foley BR (2011) Natural Genetic Variation in Social Niche Construction: Social Effects of Aggression Drive Disruptive Sexual Selection in Drosophila melanogaster. The American Naturalist 177: 645–654. 10.1086/659631 21508610

[13] Wolf Jochen BW, Traulsen A, James R (2011) Exploring the Link between Genetic Relatedness r and Social Contact Structure k in Animal Social Networks. The American Naturalist 177: 135–142. 10.1086/657442 21117963

[14] Sallet J, Mars RB, Noonan MP, Andersson JL, O'Reilly JX, et al (2011) Social Network Size Affects Neural Circuits in Macaques. Science 334: 697–700. 22053054 10.1126/science.1210027

[15] Sueur C, Petit O, De Marco A, Jacobs AT, Watanabe K, et al (2011) A comparative network analysis of social style in macaques. Animal Behaviour 82: 845–852.

[16] Sih A, Hanser S, McHugh K (2009) Social network theory: new insights and issues for behavioral ecologists. Behavioral Ecology and Sociobiology 63: 975–988.

[17] Vital C, Martins EP (2011) Strain differences in zebrafish (*Danio rerio*) social roles and their impact on group task performance. Journal of Comparative Psychology 125: 278–285. 21707139 10.1037/a0023906

[18] Lusseau D (2003) The emergent properties of a dolphin social network. Proceedings of the Royal Society of London, Series B: Biological Sciences 270: S186–S188. 14667378 10.1098/rsbl.2003.0057 PMC1809954

[19] Voelkl B, Noe R (2008) The influence of social structure on the propagation of social information in artificial primate groups: A graph-based simulation approach. Journal of Theoretical Biology 252: 77–86. 18342891 10.1016/j.jtbi.2008.02.002

[20] Flack JC, Girvan M, Waal FBMd, Krakauer DC (2006) Policing stabilizes construction of social niches in primates. Nature 439: 426–429. 16437106 10.1038/nature04326

[21] King AP, White DJ, West MJ (2003) Female proximity stimulates development of male competition in juvenile brown-headed cowbirds, *Molothrus ater* . Animal Behaviour 68: 817–828.

[22] Fernandez-Juricic E, Siller S, Kacelnik A (2004) Flock density, social foraging, and scanning: an experiment with starlings. Behavioral Ecology 15: 371–379.

[23] Fewell JH (2003) Social insect networks. Science 301: 3. 10.1126/science.1088945 14512616

[24] Thornton A, Clutton-Brock T (2011) Social learning and the development of individual and group behaviour in mammal societies. Philosophical Transactions of the Royal Society B-Biological Sciences 366: 978–987. 10.1098/rstb.2010.0312 PMC3049086 21357220

[25] King AJ, Cowlishaw G (2009) Leaders, followers and group decision-making. Communicative and Integrative Biology 2: 3. 10.4161/cib.7562 PMC2686370 19513268

[26] Bode NWF, Wood AJ, Franks DW (2011) The impact of social networks on animal collective motion. Animal Behaviour 82: 29–38.

[27] Brown C, Laland K (2011) Social Learning in Fishes. Fish Cognition and Behavior: Wiley-Blackwell. pp. 240–257.

[28] McClure MM, McIntyre PB, McCune AR (2006) Notes on the natural diet and habitat of eight danionin fishes, including the zebrafish *Danio rerio* . Journal of Fish Biology 69: 553–570.

[29] Spence R, Gerlach G, Lawrence C, Smith C (2008) The behaviour and ecology of the zebrafish, *Danio rerio* . Biological Reviews 83: 13–34. 18093234 10.1111/j.1469-185X.2007.00030.x

[30] Barcellos LJG, Ritter F, Kreutz LC, Cericato L (2010) Can zebrafish *Danio rerio* learn about predation risk? The effect of a previous experience on the cortisol response in subsequent encounters with a predator. Journal of Fish Biology 76: 1032–1038.

[31] Kelley JL, Magurran AE (2003) Learned predator recognition and antipredator responses in fishes. Fish and Fisheries 4: 216–226.

[32] Manassa R, McCormick M (2012) Social learning and acquired recognition of a predator by a marine fish. Animal Cognition 15: 559–565. 22453926 10.1007/s10071-012-0484-z

[33] Morrell LJ, Croft DP, Dyer JRG, Chapman BB, Kelley JL, et al (2008) Association patterns and foraging behaviour in natural and artificial guppy shoals. Animal Behaviour 76: 855–864.

[34] Kiflawi M, Mazeroll AI (2006) Female leadership during migration and the potential for sex-specific benefits of mass spawning in the brown surgeonfish (*Acanthurus nigrofuscus*). Environmental Biology of Fishes 76: 19–23.

[35] Lachlan RF, Crooks L, Laland KN (1998) Who follows whom? Shoaling preferences and social learning of foraging information in guppies. Animal Behaviour 56: 181–190. 9710476 10.1006/anbe.1998.0760

[36] Croft DP, Krause J, Couzin ID, Pitcher TJ (2003) When fish shoals meet: outcomes for evolution and fisheries. Fish and Fisheries 4: 138–146.

[37] Lindeyer CM, Reader SM (2010) Social learning of escape routes in zebrafish and the stability of behavioural traditions. Animal Behaviour 79: 827–834.

[38] Stewart A, Gaikwad S, Kyzar E, Green J, Roth A, et al (2012) Modeling anxiety using adult zebrafish: A conceptual review. Neuropharmacology 62: 135–143. 21843537 10.1016/j.neuropharm.2011.07.037 PMC3195883

[39] Gerlai R (2012) Using zebrafish to unravel the genetics of complex brain disorders Behavioral Neurogenetics. In: Cryan JF, Reif A, editors: Springer Berlin Heidelberg. pp. 3–24. 10.1007/7854_2011_180 22250005

[40] Pritchard VL, Lawrence J, Butlin RK, Krause J (2001) Shoal choice in zebrafish, *Danio rerio*: the influence of shoal size and activity. Animal Behaviour 62: 1085–1088. 10.1006/anbe.1998.1010 10202085

[41] Saverino C, Gerlai R (2008) The social zebrafish: Behavioral responses to conspecific, heterospecific, and computer animated fish. Behavioural Brain Research 191: 77–87. 18423643 10.1016/j.bbr.2008.03.013 PMC2486438

[42] Croft DP, Arrowsmith BJ, Bielby J, Skinner K, White E, et al (2003) Mechanisms underlying shoal composition in the Trinidadian guppy, Poecilia reticulata. Oikos 100: 429–438.

[43] Vital C, Martins EP (2009) Using graph theory metrics to infer information flow through animal social groups: a computer simulation analysis. Ethology 115: 347–355.

[44] Stephenson K, Zelen M (1989) Rethinking centrality: methods and examples. Social Networks 11: 1–37.

[45] SAS (2009) SAS for Windows. In: Institute S, editor.

[46] Pillot MH, Gautrais J, Gouello J, Michelena P, sibbald A, et al (2010) Moving together: Incidental leaders and naïve followers. Behavioural Processes 83: 235–241. 19931601 10.1016/j.beproc.2009.11.006

[47] Reebs SG (2000) Can a minority of informed leaders determine the foraging movements of a fish shoal? Animal Behaviour 59: 403–409. 10675263 10.1006/anbe.1999.1314

[48] Barzilai-Nahon K (2008) Toward a theory of network gatekeeping: A framework for exploring information control. Journal of the American Society for Information Science 59: 1493–1512.

[49] Kurvers RHJM, Eijkelenkamp B, van Oers K, van Lith B, van Wieren SE, et al (2009) Personality differences explain leadership in barnacle geese. Animal Behaviour 78: 447–453.

[50] Fichtel C, Pyritz L, Kappeler PM (2010) Coordination of group movements in non-human primates. In: Boos M, Kolbe M, Ellwart S, Kappeler PM, editors. Coordination in human and non-human primate groups. Heidelberg: Springer. pp. 37–56.

[51] Oswald M, Robison BD (2008) Strain-specific alteration of zebrafish feeding behavior in response to aversive stimuli. Canadian Journal of Zoology 86: 1085–1094. 21379405 10.1139/Z08-085 PMC3048467

[52] Johnstone RA, Manica A (2011) Evolution of personality differences in leadership. Proceedings of the National Academy of Sciences 108: 8373–8378. 10.1073/pnas.1102191108 PMC3100967 21536882

[53] Moretz JA, Martins EP, Robinson BD (2007) Behavioral syndromes and the evolution of correlated behavior in zebrafish. Behavioral ecology 18: 556–562.

[54] Reader SM, Laland KN (2000) Diffusion of foraging innovations in the guppy. Animal Behaviour 60: 175–180. 10973718 10.1006/anbe.2000.1450

[55] Miller JL, King AP, West MJ (2008) Female social networks influence male vocal development in brown-headed cowbirds, *Molothrus ater* . Animal Behaviour 76: 931–941.

[56] Katz M, Lachlan RF (2003) Social learning of food types in zebra finches (*Taenopygia guttata*) is directed by demonstrator sex and feeding activity. Animal Cognition 6: 5. 10.1007/s10071-003-0158-y 12658531

